# Innovative approaches in the treatment of chronic plantar fasciitis: comparison of pulsed radiofrequency ablation and surgical intervention

**DOI:** 10.1007/s00264-024-06261-x

**Published:** 2024-07-31

**Authors:** Celal Armağan, Zekeriya Okan Karaduman, Mehmet Arıcan, Yalcın Turhan, İlyas Kaban, Veysel Uludağ

**Affiliations:** 1 Faculty of Medicine, Department of Orthopaedics and Traumatology, Duzce University, Konuralp/Duzce, 81620 Turkey; 2 Faculty of Health Sciences, Department of Physiotherapy and Rehabilitation Düzce, Düzce University, Duzce, Turkey

**Keywords:** Functional outcomes, Pain management, Pulsed radiofrequency ablation, Surgical intervention, Chronic plantar fasciitis

## Abstract

**Purpose:**

This study aimed to compare the effectiveness of Pulsed Radiofrequency Ablation (PRFA) and surgery for treating chronic plantar fasciitis, focusing on pain relief and functional outcomes.

**Methods:**

A prospective study involved 30 patients with chronic plantar fasciitis unresponsive to 12 months of conservative treatment. Patients were divided into PRFA (*n* = 17) and surgical (*n* = 13) groups. Clinical evaluations were conducted preoperatively and at three, six and 12 months postoperatively using VAS, AOFAS, FFI, and RMS scores. Radiological measurements assessed foot structure impact.

**Results:**

Both PRFA and surgery significantly reduced pain and improved function. PRFA had a shorter operative time and quicker return to activities (*p* < 0.001). At 3 months, PRFA showed superior VAS, FFI, and RMS scores (*p* < 0.05). Long-term outcomes were similar. No major complications occurred, but minor complications were higher in the surgical group (*p* < 0.01).

**Conclusions:**

PRFA is a minimally invasive, effective treatment for chronic plantar fasciitis with quicker recovery and lower complication rates compared to surgery. Both treatments offer comparable long-term benefits. Further studies are needed to confirm these findings.

**Supplementary Information:**

The online version contains supplementary material available at 10.1007/s00264-024-06261-x.

## Introduction

Plantar fasciitis is the most common underlying cause of heel pain in patients presenting to outpatient clinics. It accounts for approximately 1% of cases seen in orthopaedic clinics [1, 2].This condition, which is a prevalent cause of heel pain, typically manifests as a sharp pain during the first steps in the morning or after prolonged periods of rest. As the day progresses and activity increases, the pain may subside, but it often returns after extended periods of standing or walking [3].

The exact cause of plantar fasciitis is not fully understood, but the biomechanics and overloading of the plantar aponeurosis are considered primary factors. Risk factors include obesity, pes planus (flat feet), pes cavus (high-arched feet), shortened Achilles tendon, and restricted ankle dorsiflexion. Additionally, environmental factors such as walking barefoot, prolonged standing, walking on hard surfaces, and the use of inappropriate footwear contribute to the development of plantar fasciitis [4]. Conservative treatments also include External Shock Wave Therapy, which has shown effectiveness in alleviating plantar fasciitis symptoms [5].

The initial treatment for plantar fasciitis typically involves non-steroidal anti-inflammatory drugs (NSAIDs) and various conservative methods. These methods include the use of heel pads and taping, therapeutic orthotic insoles, oral anti-inflammatory medications, steroid injections, stretching exercises for the Achilles tendon and plantar fascia, night splints, and physical therapy [6]. Conservative treatments usually yield successful results, with a significant portion of patients experiencing symptom relief. However, in recalcitrant cases where symptoms persist despite conservative therapy, surgical intervention may be required. Open plantar fascia release surgery is a common method for treating chronic plantar fasciitis, but it is associated with a long recovery period and potential complications [7, 8].

In recent years, radiofrequency ablation (RFA) has emerged as a minimally invasive technique for the treatment of recalcitrant plantar fasciitis. RFA aims to disrupt pain transmission by targeting nerve tissue [9]. There are two types of RFA: thermal radiofrequency ablation (TRFA) and pulse radiofrequency ablation (PRFA). TRFA involves the use of high temperatures to desensitize nerve endings, aiming to reduce pain by disrupting pain signal transmission. However, the use of high temperatures carries a risk of damaging surrounding tissues, potentially leading to side effects and complications (3). PRFA, on the other hand, utilizes low-temperature pulsed electric fields to achieve nerve ablation in a less invasive manner. PRFA’s neurodestructive effects are minimal, reducing pain without damaging surrounding soft tissues. The relatively long pauses between pulses in PRFA prevent excessive heating of nerve tissue, thus providing pain management without causing nerve damage. Compared to thermal RFA, PRFA has a minimal risk of causing neuritis, neuroma, and deafferentation pain [10–12].

The aim of this study is to compare the short-term and long-term effects of PRFA and open plantar fascia release surgery on pain and functional outcomes in patients with recalcitrant plantar fasciitis who have not responded to conservative treatments. By evaluating these two methods, we seek to determine which treatment offers better pain relief and functional improvement, thus providing a more effective and safer alternative for patients suffering from chronic plantar fasciitis. This comparison is crucial as it can guide clinical decisions and improve patient outcomes by identifying the most beneficial treatment approach for those who have not experienced relief from traditional conservative therapies.

## Methods

This prospective study was conducted at the Orthopedics and Traumatology Clinic of Düzce University Faculty of Medicine. It included 30 patients diagnosed with plantar fasciitis (PF) following clinical examination and investigations, excluding other potential causes of heel pain. Patients were included if they had been symptomatic for at least 12 months and had not responded to conservative treatments, and were over 18 years old. The patients were divided into two groups: the PRFA group (*n* = 17), which underwent pulse radiofrequency ablation, and the surgical group (CR group, *n* = 13), which underwent open plantar fascia release surgery. Detailed information about the study’s purpose, procedure, duration, potential complications, and possible problems were provided to all participants Patients were randomly assigned to the treatment groups without considering smoking status. Ethical approval for the study was obtained from the Ethics Committee of Düzce University Faculty of Medicine on March 26, 2023, with decision number 2023/43.

Inclusion criteria for the study were: patients over 18 years of age with symptoms for at least 12 months, diagnosed with plantar fasciitis through clinical and radiographic differential diagnosis, and unresponsive to first-line exercises, standard insoles, and non-steroidal anti-inflammatory treatment. Exclusion criteria included previous heel surgery, steroid injection into the heel within the last three months, history of heel trauma, allergic reactions to anesthetic agents, bone anomalies in the knee and ankle, local infections, presence of a pacemaker, peripheral neuropathy, and malignancy.

Patients presenting to the clinic with heel pain, diagnosed with PF through history and physical examination, symptomatic for at least 12 months, and unresponsive to at least six months of conservative treatments (exercise, standard insoles, NSAIDs, ESWT, and local steroid injections) were evaluated. Detailed examinations were performed, and differential diagnoses were made using radiographs, MRI, CT scans, blood tests, and other investigations as needed. Sociodemographic characteristics and complaints of the patients were recorded. Hemogram and biochemical tests were performed, and lateral and anterior-posterior foot radiographs were taken. Patients were advised to continue their standard exercise programs and refrain from using NSAIDs during the study period. Clinical evaluations were conducted preoperatively and postoperatively at three months, six months, and one year, using the Foot Function Index (FFI), American Orthopaedic Foot & Ankle Society (AOFAS) ankle-hindfoot score, Visual Analog Scale (VAS), and Roles-Maudsley Score (RMS), along with lateral and anterior-posterior foot radiographs.

### Clinical evaluation methods

#### Foot function index (FFI)

The FFI is a commonly used self-report form that measures the impact of foot pathologies on pain, disability, and activity limitation. Patients rate their pain on a scale from 0 (no pain) to 10 (worst possible pain) across three sections: pain, disability, and activity limitation. Each section is scored and averaged to a total score out of 100, with higher scores indicating greater pain, disability, and activity limitation [13].

#### American orthopaedic foot & ankle society (AOFAS) score

This score evaluates functional outcomes objectively, including pain (40 points), limitations in activities (50 points), walking distance and gait abnormalities, range of motion, and stability of the foot (10 points). Scores range from 0 to 100, with 91–100 considered excellent, 81–90 good, 71–80 fair, and 70 and below poor [14].

#### Visual analog scale (VAS)

The VAS is a simple and widely used method for assessing pain intensity. Patients rate their pain on a scale from 0 (no pain) to 10 (worst possible pain). In this study, patients evaluated their pain at rest, during the first step in the morning, and during exercise [15].

#### Roles-maudsley score (RMS)

The RMS is a practical and reliable method for assessing pain and its relationship with activity. It is easy to understand for patients and is rated on a four-point scale: (1) no pain during all activities (excellent result), (2) occasional discomfort during all activities (good result), (3) pain after prolonged activity (fair result), and (4) pain limiting daily activities (poor result) [16].

### Radiological evaluation methods

#### Calcaneal pitch angle

The angle between a line drawn parallel to the inferior cortex of the calcaneus and a line drawn from the lowest point of the calcaneus to the lowest point of the fifth metatarsal head. This angle reflects the inclination of the calcaneus relative to the horizontal plane, with a normal value of 21–29 degrees. It decreases in equinus and increases in calcaneus deformity [17].

#### Meary’s angle

The angle between the long axis of the talus and the long axis of the first metatarsal. This angle indicates the alignment of the forefoot relative to the hindfoot, with a normal range of -4° to + 4°. Angles of 4–15 degrees indicate mild, 15–40 degrees moderate, and over 40 degrees severe pes planus [18].

#### Hibbs angle

The angle between the longitudinal axis of the first metatarsal and the longitudinal axis of the calcaneus, normally 130–160 degrees [19].

#### AP talus-first metatarsal angle

The angle between the long axis of the talus and the long axis of the first metatarsal, normally ranging from − 5 to + 5 degrees [20].

#### AP talocalcaneal angle (kite angle)

The angle between the long axis of the talus and the long axis of the calcaneus, with a normal range of 15–30 degrees [20].

### Treatment methods

#### Pulse radiofrequency ablation (PRFA)

**Fig. 1:**
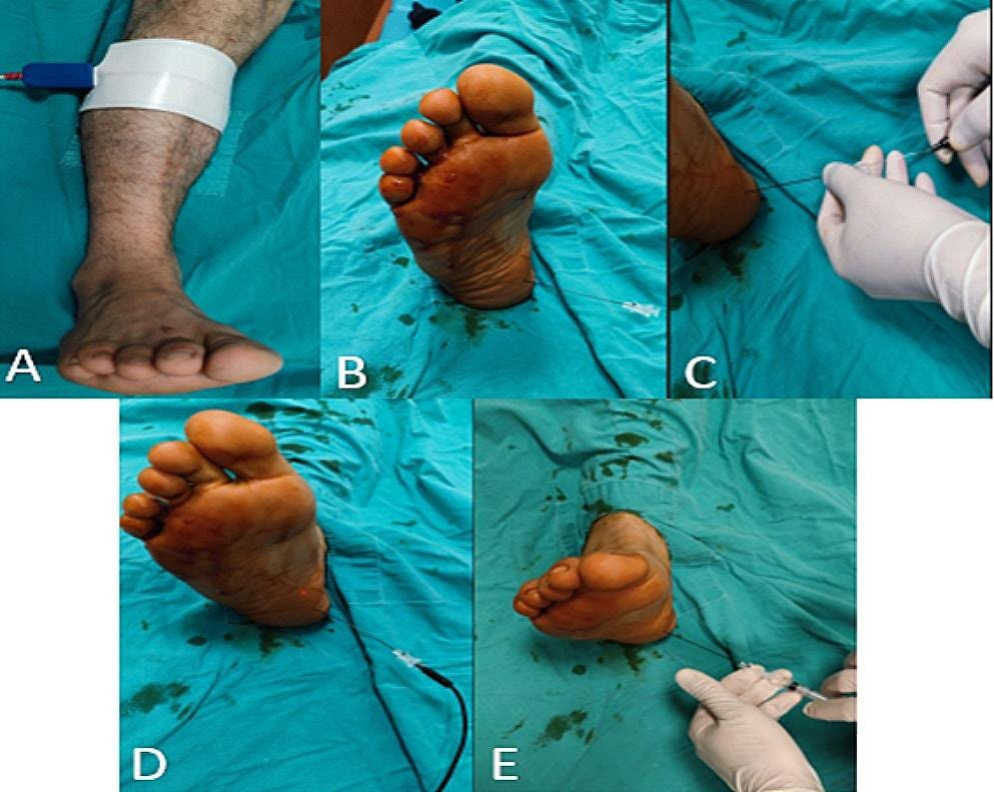
Stages of pulsed radiofrequency ablation. **A**: Placement of the grounding pad **B**: Application of hypodermic cannula with a stylet **C**: Removal of the stylet needle and insertion of the electrode **D**: Application of radiofrequency **E**: Administration of anesthetic substance after the procedure

Procedures were performed in the operating room at Duzce University Faculty of Medicine. Patients were marked at the service, and a grounding pad was placed on the calf of the treated extremity. A hypodermic cannula with a stylet was inserted through the skin to the anteromedial calcaneus. The stylet was removed, and an electrode was placed. Using the COOLIEF radiofrequency device, simulation mode was activated, and current was gradually increased from 0 to 1 V to determine the sensory threshold. If the threshold was above 1 V, the application area was adjusted. Sensory response was confirmed at voltages below 0.5 volts. Motor control was conducted at 2 Hz, with no involuntary contractions indicating proper placement. PRFA was applied at 42 °C for eight minutes at 20 m/s intervals, aiming for an impedance of 250–500 ohms. After the procedure, 1 ml of 5 mg bupivacaine was injected to prevent pain during nerve ablation (Fig. 1) [21].

#### Open plantar aponeurosis release

**Fig. 2:**
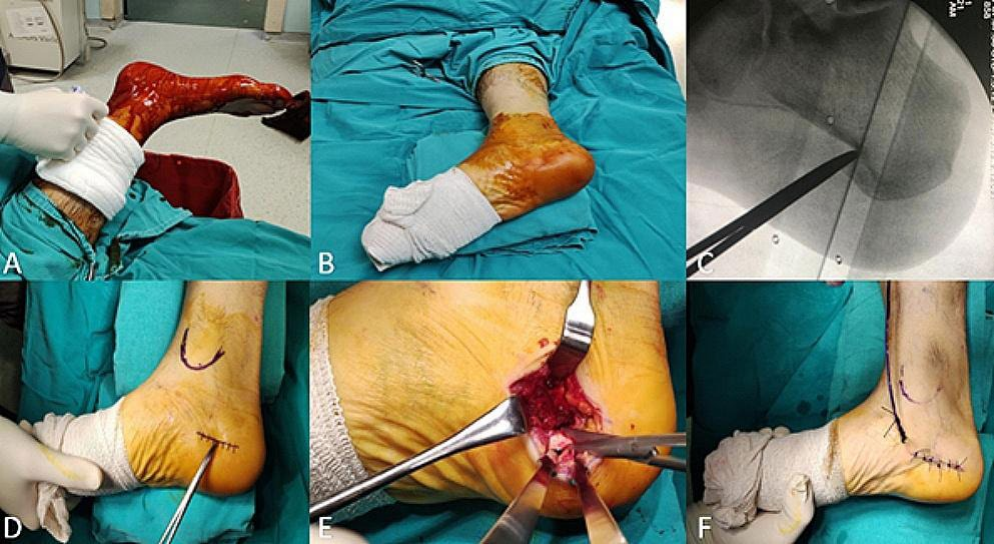
Stages of plantar fascia release surgery. **A**: Sterilization of the operation site **B**: Patient position and view after covering with sterile drapes **C**: Identification of the incision area with fluoroscopy **D**: Display of the incision area **E**: Exposure and release of the plantar fascia **F**: Placement of the drain and skin closure

Procedures were performed in the operating room with the patient under spinal anaesthesia. The affected extremity was positioned laterally, sterilized, and draped. A 3 cm incision was made medially from the heel, and approximately 40% of the plantar fascia was released. The wound was irrigated, a drain was placed, and the incision was sutured and dressed (Fig. 2) [22].

### Statistical analysis

Statistical analyses were performed using NCSS 2007 Statistical Software (Utah, USA). Descriptive statistical methods (mean, standard deviation, median, interquartile range) were used, along with the Shapiro–Wilk normality test. Independent t-tests compared normally distributed variables, Friedman tests for multiple time comparisons, Dunn’s multiple comparison tests for subgroup comparisons, Wilcoxon tests for paired time comparisons, Mann Whitney U tests for non-normally distributed variables, and chi-square tests for categorical data. Results were considered significant at *p* < 0.05.

## Results

This section presents the findings of the study, including demographic characteristics, clinical outcomes, and radiological measurements of the participants in both the surgery and PRF groups. The results are categorized into three main tables that highlight the key comparisons and statistical analyses performed.

**Table 1:** Demographic characteristics and chronic diseases

Variable	Surgery Group (*n* = 13)	PRF Group (*n* = 17)	*p* Value
Age (Mean ± SD)	48.46 ± 3.8	47.76 ± 6.86	0.745
Gender			0.087
Male	5 (38.5%)	2 (11.8%)	
Female	8 (61.5%)	15 (88.2%)	
BMI (Mean ± SD)	32.59 ± 4.15	35.14 ± 6.44	0.225
Education Level			0.524
Primary School	6 (46.2%)	11 (64.7%)	
High School	5 (38.5%)	5 (29.4%)	
University	2 (15.4%)	1 (5.9%)	
Occupation			0.174
Housewife	3 (23.1%)	9 (52.9%)	
Civil Servant	1 (7.7%)	2 (11.8%)	
Worker	7 (53.9%)	6 (35.3%)	
Self-employed	2 (15.4%)	0 (0%)	
Smoking Status			0.269
No	8 (61.5%)	7 (41.2%)	
Yes	5 (38.5%)	10 (58.8%)	
Diabetes			0.091
No	7 (53.9%)	14 (82.4%)	
Yes	6 (46.2%)	3 (17.7%)	
Hypertension			0.794
No	9 (69.2%)	11 (64.7%)	
Yes	4 (30.8%)	6 (35.3%)	
Goiter			0.368
No	11 (84.6%)	12 (70.6%)	
Yes	2 (15.4%)	5 (29.4%)	
Asthma			0.014
No	9 (69.2%)	17 (100%)	
Yes	4 (30.8%)	0 (0%)	
Comorbidities			0.558
No	4 (30.8%)	7 (41.2%)	
Yes	9 (69.2%)	10 (58.8%)	

*: significant difference p˂0.05. SD: Standard deviation. BMI: Body mass index. PRF: Pulse Radiofrequency Ablation

Based on the data in Table 1, there were no significant differences between the surgery and PRF groups in terms of age, gender, BMI, education level, occupation, smoking status, and most chronic diseases. However, the prevalence of asthma was significantly lower in the PRF group (*p* = 0.014), indicating a notable difference in asthma rates between the groups. For all other variables, p values were greater than 0.05, indicating no statistically significant differences between the surgery and PRF groups

**Table 2:** Combined FFI, AOFAS, VAS Exercise, and roles Maudsley scores

Variable	Time Point	Surgery Group (*n* = 13)	PRF Group (*n* = 17)	*p* Value
FFI Disability (Mean ± SD)	Baseline	85.31 ± 10.84	88.24 ± 9.3	0.438
	3 Months	56.85 ± 16.75	27.76 ± 29.75	0.018*
	6 Months	22.15 ± 16.72	20.18 ± 25.15	0.604
	1 Year	3.46 ± 4.86	17.94 ± 29.56	0.737
FFI Activity Limitation (Mean ± SD)	Baseline	46.31 ± 18.71	42.35 ± 13.93	0.801
	3 Months	49.23 ± 18.77	10.71 ± 13.42	0.001*
	6 Months	10.31 ± 11.19	7.53 ± 9.91	0.401
	1 Year	1.38 ± 2.76	7.29 ± 11.22	0.245
AOFAS (Mean ± SD)	Baseline	41.38 ± 4.91	43.71 ± 8.17	0.613
	3 Months	69.08 ± 9.62	86.18 ± 12.34	0.002*
	6 Months	89.15 ± 8.09	92.29 ± 10.78	0.237
	1 Year	95.54 ± 6.6	92.47 ± 11.31	0.930
VAS Exercise (Mean ± SD)	Baseline	9.77 ± 0.44	9.47 ± 0.72	0.245
	3 Months	6 ± 2.16	2.76 ± 2.97	0.007*
	6 Months	1.85 ± 1.63	2.06 ± 2.88	0.598
	1 Year	0.54 ± 0.78	1.94 ± 2.93	0.577
Roles Maudsley (Mean ± SD)	Baseline	3.92 ± 0.28	3.88 ± 0.33	0.717
	3 Months	3.31 ± 0.63	1.88 ± 0.86	0.001*
	6 Months	1.69 ± 0.63	1.53 ± 0.72	0.403
	1 Year	1.08 ± 0.28	1.53 ± 0.8	0.07

*: significant difference p˂0.05. SD: Standard deviation. FFI: Foot Function Index AOFAS: American Orthopaedic Foot & Ankle Society Score. VAS: Visual Analog Scale. PRF: Pulse Radiofrequency Ablation

As illustrated in Table 2, the PRF group achieved significantly better results than the surgery group in terms of FFI disability (*p* = 0.018), FFI activity limitation (*p* = 0.001), AOFAS (*p* = 0.002), VAS exercise (*p* = 0.007), and Roles Maudsley scores (*p* = 0.001) at the three month follow-up. These p values indicate that the PRF group performed significantly better on these measures compared to the surgery group at this time point. At the 6-month and 1-year follow-ups, no significant differences were observed between the two groups (*p* > 0.05), suggesting that long-term outcomes were similar for both treatments

**Table 3:** Radiological measurements

Variable	Time Point	Surgery Group (*n* = 13)	PRF Group (*n* = 17)	*p* Value
L-Kalkaneal Pitch (Mean ± SD)	Preoperative	19.62 ± 4.29	17.76 ± 4.29	0.247
	Postoperative	17.46 ± 5.16	17.35 ± 4	0.751
L-Meary (Mean ± SD)	Preoperative	4.38 ± 2.66	4.18 ± 2.48	0.799
	Postoperative	4.38 ± 2.5	4.47 ± 3.28	0.735
L-Hibbs (Mean ± SD)	Preoperative	48.08 ± 6.24	43.24 ± 7.9	0.166
	Postoperative	47.23 ± 5.34	42.59 ± 7.49	0.065
AP talus-1st metatarsal angle (Simmon angle) (Mean ± SD)	Preoperative	7.23 ± 4.4	5.29 ± 4.12	0.158
	Postoperative	6.15 ± 3.6	4.53 ± 2.94	0.227
AP-Talokalkaneal (Mean ± SD)	Preoperative	26.77 ± 11.16	27.06 ± 6.94	0.900
	Postoperative	26.85 ± 11.61	27.06 ± 5.73	0.883

*: significant difference p˂0.05. SD: Standard deviation. L: Lateral AP: Anterı̇oposterı̇or. PRF: Pulse Radiofrequency Ablation

From the information provided in Table 3, there were no significant differences in most radiological measurements between the surgery and PRF groups at preoperative and postoperative time points. Measurements such as L-Kalkaneal Pitch, L-Meary, L-Hibbs, AP talus-first metatarsal angle (Simmon angle), and AP-Talokalkaneal angle showed no statistically significant differences between the groups (*p* > 0.05). These results indicate that the radiological outcomes were similar for both the surgery and PRF groups

## Discussion

In this study, we prospectively evaluated the effects of pulse radiofrequency ablation (PRFA) and open plantar fascia release surgery on pain and functional outcomes in patients with plantar fasciitis (PF) unresponsive to conservative treatments. Our findings indicated no significant demographic differences between the PRFA and surgery groups, but the PRFA group exhibited faster recovery in the early stages. This rapid recovery can be attributed to the minimally invasive nature of PRFA and its reduced impact on surrounding tissues.

Plantar fasciitis is a prevalent cause of heel pain, significantly impairing patients’ quality of life. The results of our study indicate that pulsed radiofrequency nerve ablation (PRFA) is an effective treatment modality for recalcitrant plantar fasciitis, offering substantial pain relief and functional improvement [23]. This aligns with the findings of Kumarendran Kanesen et al., [24] who demonstrated that PRFA is a secure and effective method for alleviating pain associated with chronic plantar fasciitis, particularly due to its minimally invasive nature and quick onset of analgesia. Additionally, our study showed that PRFA provides consistent benefits across various demographic factors, reinforcing its potential as a uniform treatment option. These findings highlight PRFA as a promising alternative for patients unresponsive to conservative treatments, emphasizing its value in managing this challenging condition effectively. Our study’s results are consistent with the literature, supporting PRFA as a valuable therapeutic approach for recalcitrant plantar fasciitis.

A study assessing the effects of radiofrequency controlled ablation with platelet-rich plasma injection in patients with refractory plantar fasciitis found that 67.4% of patients perceived themselves as fully recovered, 23.3% as partially recovered, and 9.3% as not recovered. Post-procedure patient satisfaction was high, with significant pain reduction observed [25]. Similarly, our results showed high patient satisfaction and low complication rates in the PRFA group, which can be explained by the minimally invasive nature and rapid recovery associated with PRFA. In another study evaluating long-term outcomes after plantar fasciotomy, the majority of patients reported long-term satisfaction, and 74% would choose the same procedure again. However, 44% of patients continued to experience postoperative swelling and tenderness [26].These findings align with our study, which demonstrated that PRFA has lower complication rates compared to surgery. The reduced invasiveness of PRFA, leading to less tissue damage, may account for these lower complication rates.

Conventional radiofrequency ablation has been shown to reduce pain in patients with chronic heel pain, which is more likely due to the inflammation of the plantar fascia rather than the heel spur itself [27]. This finding supports the effectiveness of PRFA in pain management observed in our study. The targeted effect of PRFA on nerve tissue likely plays a crucial role in reducing pain intensity.

A systematic review and network meta-analysis of the literature evaluated various surgical interventions for plantar fasciitis, including open and endoscopic plantar fasciotomy, gastrocnemius release, radiofrequency microtenotomy and dry needling. The study concluded that all surgical options provided short to medium-term relief with improvements in VAS and AOFAS scores and no significant complications. However, the review emphasized the need for larger randomized trials to determine long-term outcomes and identify the most effective surgical treatment. These findings are in line with our study, which also demonstrated the efficacy of percutaneous plantar fasciotomy in providing significant symptomatic relief without altering the medial longitudinal arch of the foot [28].

In the literature, the effectiveness of RFA therapies for managing persistent pain syndromes has been widely examined. Techniques such as genicular nerve radiofrequency ablation for knee osteoarthritis, nerve ablation for foot and ankle pain, and ultrasound-guided ablation for sural neuralgia have shown successful outcomes in various anatomical regions [29–31]. These methods have proven particularly effective in cases where conservative treatments have failed, providing significant pain relief and functional improvement. The novelty of our study lies in evaluating the application and long-term effects of these techniques specifically in the treatment of recalcitrant plantar fasciitis. By assessing the effectiveness of radiofrequency ablation in comparison to other treatment methods for plantar fasciitis, our study contributes valuable insights to the clinical management of this condition. These findings, supported by further large-scale randomized studies, will help establish the long-term outcomes and clinical relevance of these treatment approaches.

This study has several limitations. Firstly, it was conducted at a single center, which limits the generalizability of the results to broader populations. Secondly, long-term outcomes have not yet been evaluated, so further follow-up is needed to fully assess the long-term efficacy of PRFA and surgery. Additionally, individual differences in patients’ recovery processes and responses to treatment should be considered. Finally, the sample size of our study is limited, and larger-scale studies are necessary.

## Conclusion

In this study, we compared the effectiveness of pulse radiofrequency ablation (PRFA) and surgery in patients with plantar fasciitis unresponsive to conservative treatments. PRFA was found to be superior to surgery due to its neurodestructive effect, which leads to significant pain relief, shorter operation time, and lower complication rate. In the long term, both treatment methods showed similar efficacy. PRFA is considered an effective and reliable option for the treatment of plantar fasciitis. These findings suggest that PRFA should be considered as an alternative to surgery in clinical practice. Future large-scale and long-term studies are needed to better understand the efficacy and safety profiles of these treatment methods. This study contributes significantly to the development of innovative and effective methods for the treatment of plantar fasciitis.

## Electronic supplementary material

Below is the link to the electronic supplementary material.

Supplementary Material 1

## Data Availability

The datasets used and/or analyzed during the current study available from the corresponding author on reasonable request.
