# Supplementary material for: Innovative approaches in the treatment of chronic plantar fasciitis: comparison of pulsed radiofrequency ablation and surgical intervention
Source: Int Orthop. 2024 Jul 31;48(10):2719–26. doi: 10.1007/s00264-024-06261-x (PMC11422293; doi:10.1007/s00264-024-06261-x)
Supplement: Supplementary file 1 — Supplementary Material 1 [file 264_2024_6261_MOESM1_ESM.docx]

30/05/2024

Dear Editor,

We would like to submit our manuscript entitled **Innovative Approaches in the Treatment of Chronic Plantar Fasciitis: Comparison of Pulsed Radiofrequency Ablation and Surgical Intervention** for your consideration as a publication in **International Orthopaedics**. The manuscript is original, all the authors were active participants, and the manuscript is not currently under consideration for publication in another journal. All authors gave consent for submission of the manuscript. The study has ethics approval as stated in materials and methods section. The authors declare that they have no conflict of interest.

Thank you for your kind cooperation.

Sincerly yours, Z. Okan Karaduman,MD,Associate Professor

**Corresponding author:** Z. Okan Karaduman MD, Associate Professor

School of Medicine, Duzce University; Department of Orthopedics and Traumatology, 81620, Konuralp/Duzce, Turkey.

**E-mail:** [okankaraduman@duzce.edu.tr](mailto:okankaraduman@duzce.edu.tr)

**Orcid:** 0000-0002-6719-3666

**Phone:** +90 380 542 13 90 / 6552, +90 5055753830 (mobile phone)
